# A case study comparing the detection ability of cadaver detection dogs and electronic nose technology to a clandestine grave

**DOI:** 10.1371/journal.pone.0360233

**Published:** 2026-10-21

**Authors:** Amber O. Brown, Sandali Alahakone, Erin Lamb, Daniel Pasin, Darryl Hawker, Taoping Liu, Wentian Zhang, Shari L. Forbes, Steven Su, Maiken Ueland

**Affiliations:** 1 Centre for Forensic Sciences, School of Mathematical and Physical Sciences, University of Technology Sydney, Sydney, New South Wales, Australia; 2 University of North Florida, Jacksonville, Florida, United States of America; 3 Hyperion Data, Griffith, New South Wales, Australia; 4 NSW Police Force Canine Unit, Parramatta, New South Wales, Australia; 5 School of Information Mechanics and Sensing Engineering, Xidian University, Xi’an, China; 6 School of Mathematical and Physical Science, University of Technology Sydney, Sydney, New South Wales, Australia; 7 Department of Chemistry and Biochemistry, University of Windsor, Windsor, Ontario, Canada; 8 College of Medical Information and AI, Shandong First Medical University, Jinan, Shandong, China; Shuguang Hospital Affiliated to Shanghai University of Traditional Chinese Medicine, CHINA

## Abstract

Cadaver detection dogs (CDDs) are one of the best tools to locate clandestinely buried human remains. However, large data gaps exist in what VOC classes and concentrations are targeted by CDDs for detection. This study compared the alert responses produced by CDDs used by the New South Wales Police Force to responses generated by an electronic nose (named the NOS.E) with metal oxide sensors to a clandestine burial. For confirmatory analysis, VOC samples of the clandestine burial were taken, and compounds were identified using GC × GC-TOFMS. While statistical work was limited due to sample sizes, inferences were made about VOC presence and concentration to NOS.E and detection dog ability. Both CDDs and NOS.E were able to detect targeted chemical classes (sulfur and nitrogen containing compounds, alcohols, alkanes and aldehydes) which was supported by GC × GC-TOFMS. Correlation analyses demonstrated non-significant, but observational patterns that CDDs true positive alerts were associated with higher NOS.E responses to sulfur and nitrogen containing compounds, alcohols and alkanes. Additionally, a “no alert” or “False Positive” alert was associated with lower NOS.E responses. This work supports that it is both the presence and abundance of VOC chemical classes that influence true positive alerts from CDDs to buried human remains.

## 1. Introduction

Locating long-term missing persons (missing longer than three months) [1] poses some of the most resource-intensive and challenging obstacles to law enforcement. The first step of every missing person’s investigation is commencing physical searches in areas where the subject was last observed. If there is concern that the missing individual is deceased, law enforcement will incorporate methods for detecting and recovering human remains. When a body is exposed to the elements or left outdoors, remains can become scattered or buried due to environmental changes or animal activity. However, when a body is deliberately disposed of by someone attempting to conceal evidence of a crime, remains may be buried at various depths in sediment [2]. The recovery of these victims can be challenging with methods relying on visually locating remains (*e.g.,* ground or aerial searches) and may require additional methods (*e.g.,* detection of decomposition odor) for their location and retrieval. Cadaver detection dogs (CDDs) are currently one of the best tools to locate human remains that are obscured by environmental (*i.e.,* soil, water and vegetation) factors. CDDs have a remarkable olfactory system [3–5] and are trained to detect the specific volatile organic compounds (VOCs) that are produced and emitted as a result of the breakdown of macromolecules (*e.g.,* proteins, lipids and carbohydrates) during decomposition [6–8]. VOCs have a high vapor pressure and low molecular weight that allow for continuous emission into the environment [9,10] either endogenously through the victim, or exogenously from their perfumes, lotions or health products [11].

VOCs disperse through the environment from an area of high concentration (*i.e.,* the “source”) to an area of low concentration. As these VOCs are dispersed continuously from a source, a “scent cone” is formed, whereby VOCs are more heavily concentrated in the air directly above the target and are diluted from the source due to environmental factors, such as wind [3]. Consequently, CDDs are able to detect trace VOCs from the edge of the scent cone and track the scent to the area where the VOCs are most concentrated [3,8,12–14]. Once CDDs locate the target, they will alert their handler through a trained behavior or signal. The detection of the scent cone by CDDs is complicated by buried remains, as soil composition and moisture content act as a sink, trapping VOCs and impacting scent cone dispersal [15,16].

To date, quantitative knowledge surrounding the types and concentrations of VOCs being dispersed from a scent cone are limited. The diffusion rate of each VOC differs due to specific chemical characteristics (*e.g.,* size, polarity, stability and reactivity) and environmental conditions present at the time of dispersal. Furthermore, it is unknown how diffusion impacts the VOC profile of a scent cone from its source (*i.e.,* an incomplete decomposition profile) and how diluted or incomplete decomposition profiles impact CDD efficacy. Current methods used to identify and quantify VOCs being produced from decomposing human remains rely on laboratory-based analytical techniques [10,15,17–21], though recent work has investigated the potential of using electronic noses (e-noses) for real-time detection of VOCs associated with human decomposition [22–24]. In addition, e-noses may be leveraged to provide insight into CDD detection ability and aid in training. In these cases, the e-nose could be deployed during training sessions to reduce false positive (reacting when there is no target) and negative (not reacting to a target) alerts of CDDs.

E-noses are portable, or semi portable, devices that mimic mammalian olfaction using multi-sensor (*e.g.,* MOX) arrays. When the sensor comes into contact with target VOCs, the electrical resistance of the sensor is lowered, increasing the voltage of the current, generating quantitative signals that are impacted by the concentration of specific VOC classes. In an outdoor environment, the baseline response of the sensor is impacted by reference air naturally containing ambient VOCs. In order for an alert to be recorded, the target VOCs must be at a higher concentration than those found ambiently [23]. For scenarios where e-noses will be used to detect human remains, care must be taken in selecting MOX sensors, as the prevalence and abundance of targeted chemical classes change over the course of decomposition (*i.e.,* “stages”). For robust detection, sensors targeting nitrogen-containing and sulfur-containing compounds as well as alcohols and aldehydes are a priority, as these are found in all stages of human decomposition [25].

To date, there is no standardized method for training and analyzing accuracy of CDDs in real world scenarios [7,12,26,27]. Furthermore, there is a distinct lack of data related to the detection ability of CDDs, including detection limits and quantitative analytical data relating to CDD efficiency and accuracy. For example, it is unknown whether it is the presence or concentration of specific VOCs that signal a CDD to alert. Despite years of research into the formation and emittance of decomposition VOCs, and the establishment of VOCs emitted at different stages of decomposition, little information exists as to which specific VOCs CDDs are targeting at specific timeframes to locate deceased individuals. It is currently unknown if singular VOCs are targeted, a combined VOC profile, or relative concentrations of specific VOCs or VOC classes are being tracked and are responsible for true positive CDD alerts. The lack of this information complicates training and evaluation of CDDs and has reduced CDD evidentiary capacity in court settings [28]. Both CDD training and search events and associated resources could be strengthened from secondary confirmation using e-nose technology to provide analytical support.

This study aimed to compare the response given by NOS.E, an e-nose developed in house at the University of Technology Sydney (UTS) to the alerts of the CDDs used by the New South Wales Police Force (NSWPF) when locating a clandestine burial. Specifically, the study was set up so that the responses of an electronic nose could be compared to the responses of three certified CDDs over a single, buried deceased human. For analytical confirmation (*i.e.,* identifying the chemical classes coming from buried remains), headspace from the buried remains were taken and analyzed via GC × GC-TOFMS (S1 Fig). The trial was set up to mimic a long-term missing person scenario, whereby the body was buried for approximately three months.

## 2. Materials and methods

### 2.1 Experimental site

The study was undertaken at the Australian Facility for Taphonomic Experimental Research (AFTER) located in Western Sydney, NSW. The facility encompasses approximately 4.86 hectares of Cumberland dry sclerophyll forest owned by the UTS and complies with all ethical and legal requirements to conduct research on human cadavers (UTS HREC REF NO. ETH18–2999). The donor was acquired through the UTS Body Donation Program, managed by the Surgical and Anatomical Science Facility (SASF). In accordance with NSW Anatomy Act (1977) consent was provided by all donors to use their remains for the purpose of research at AFTER. The donor was a male, aged 79, weighing 75.2 kg at the time of death (20^th^ March 2020). Prior to placement, the body was stored in a cold storage facility. On the 25^th^ of March 2020, the donor (unclothed) was placed in a supine position in a grave approximately 60 cm deep. The grave was located over 200 m away from any other human remains at the facility. After the initial placement, the grave was left undisturbed for 98 days before sampling commenced to simulate a long-term clandestine grave, in order to follow the definition of a long-term missing person (*i.e.,* over three months) [1]. The grave was sectioned into 6 regions: R1, R2, R3, R4, R5 and R6 (Fig 1). On occasions where the graves were probed (all days apart from the initial search on Day 98), 14 locations (Fig 1) within the grave were probed approximately 20–30 cm deep to allow air to escape. As quality control, and to ensure CDDs were not alerting to visual cues of disturbed soil as opposed to decomposition VOCs, three empty control graves were also prepared on the day the donor was buried and probed in the same manner as the human grave. One empty grave was selected as the control for the chemical analysis (GC × GC-TOFMS and NOS.E). A Hobo Weather Station equipped with a Hobo U30 No Remote Communication (OneTemp pty ltd. Australia) data logger was used to monitor temperature (°C), solar radiation (W/m²), relative humidity (%), wind speed (m/s) wind direction and rainfall (mm) at an hourly rate. The aforementioned environmental parameters were all assessed in relation to NOS.E responses in each trial for both pre- and post-probe scenarios.

**Fig 1:**
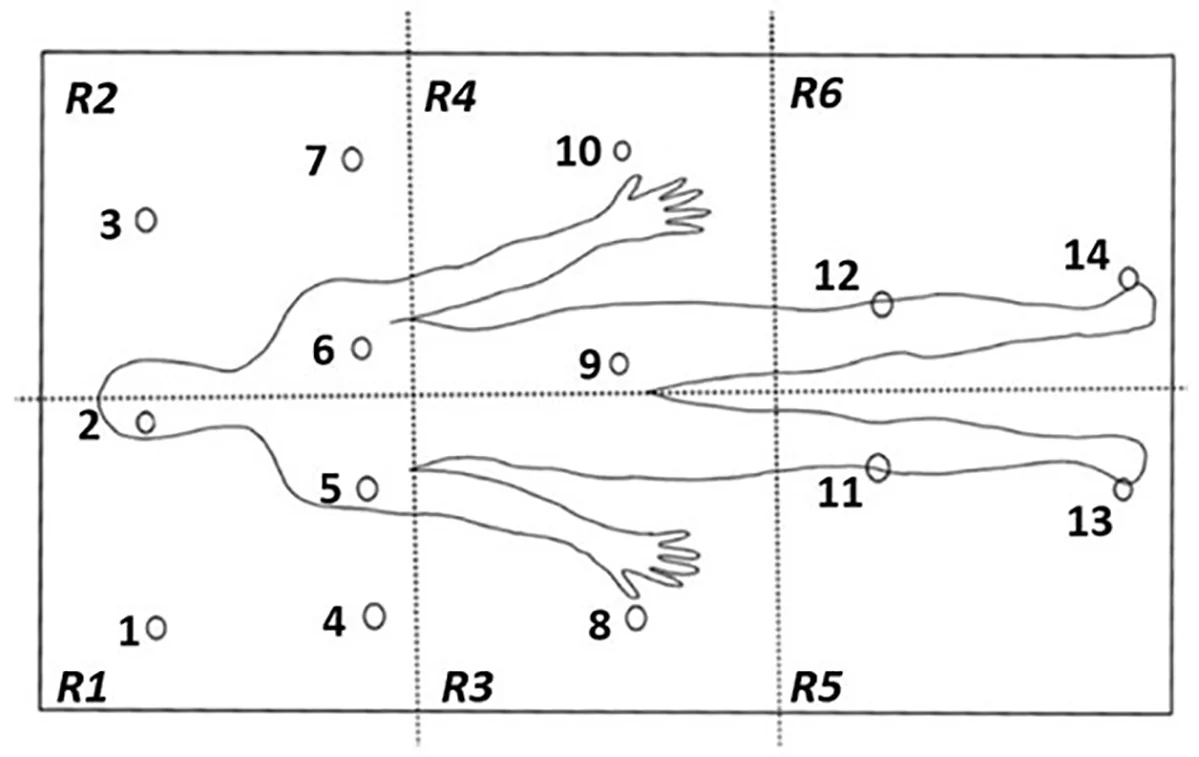
Diagram of grave site with regions and probe location numbers. Six separate search days using both the CDDs and the NOS.E were conducted on 98, 105, 120, 126, 147 and 161-days postmortem.

### 2.2 Cadaver detection dogs

At the time of this study, three CDDs accredited with the NSWPF canine unit were used (Table 1). These dogs had previously been trained on human bone, decomposition fluid and decomposition fluid-stained clothing. Throughout the study, the number of dogs present during each sampling day varied due to the operational commitments of the CDDs and their handlers. This trial was set up as a single-blind study as conducting a double-blind study using the same grave sites was not possible and would remove the required handler expert oversight, resulting in challenges in recording dog responses. The study design followed standard training protocol by the NSWPF canine unit. During this work, three individual CDDs with three respective handlers that were blind to the location of the buried remains for the first trial were used. During each trial run, each CDD conducted the search independently and without guidance from their handler. The primary trainer was not blind to the grave location and supervised the CDD detection responses from afar to determine whether a definitive alert was presented. Alerts were recorded when the CDD entered the known GPS location of the grave and had an opportunity to alert. The alerts were defined as “True Positive”, where the CDD alerted to an area that had a NOS.E sensor response above baseline, “True Negative”, where the CDD did not alert to an area that did not have a NOS.E sensor response above baseline, “False Positive”, where the CDD alerted to an area that did not have a NOS.E sensor response above baseline, and “False Negative” where the CDD did not alert to an area where the NOS.E had a sensor response above baseline. All alerts were recorded (“True Positive”, “True Negative”, “False Positive”, and “False Negative”) after the confirmation from the expert opinion of the primary trainer as well as confirmatory NOS.E signal responses above baseline. The evaluation of the dogs was performed following animal ethics approval (ETH20–5205).

**Table 1:** Summary of the biological and training information associated with each cadaver detection dog assessed at the time of the study.

ID	Age (years)	Breed	Experience at time of study (years)	No. of Trials Present
**Dog 1**	3	English Springer Spaniel	Approx. 1	1/6
**Dog 2**	4	Labrador Retriever	Approx. 2	4/6
**Dog 3**	7.5	English Springer Spaniel	Approx. 6	4/6

During the search events, each dog was taken into the site along the perimeter (the starting location differed between site visits), away from any existing donors on site, released off lead approximately 150 m from the grave site and was allowed to conduct a wide search of the area. The dogs were rewarded with a tennis ball (play reward) if they were able to correctly locate the grave as per their training. As sampling weeks continued, there was a chance that CDDs grew familiar with the area and the location of the grave. Therefore, in order to prevent false positive alerts, the dogs were let off leash from different directions each sampling event. If multiple dogs were present during a sampling event, there was a concern that a CDD may recognize another dog’s scent over the grave and make a false positive alert. To combat this, the handlers would guide the dogs over each of the three control graves once their run was completed to ensure that CDD scent was not concentrated in one location. The canine run order was also randomized each sampling time and there were breaks between the first dog and subsequent dog runs. Three control graves were selected to reduce CDD bias, but only one control grave was selected for analytical assessment to save resources.

### 2.3 Electronic nose (NOS.E) sampling

The electronic nose (NOS.E) used in this study was developed in collaboration between the UTS Centre for Forensic Science and the UTS School for Biomedical Engineering. A total of 5 Tin Oxide Gas Sensor (TGS) metal oxide (MOX; Figaro, Japan) gas sensors (Table 2) were selected and configured based on previous human decomposition studies [22,25,29–34]. The sensors were specifically selected for human decomposition VOCs and the detection limits of the sensors configured in the NOS.E were previously established [24]. Sensors selected for this study targeted alcohols (Sensors 1, 2) and alkanes (Sensors 1–3) along with nitrogen and sulfur containing compounds (Sensors 4, 5). Sensor detection limits were not assessed due to the variability of the outdoor environment. However, positive and negative cross reactivities and VOC inhibition were addressed through baseline assessment and through establishing threshold criteria for peak assessment [22].

**Table 2:** NOS.E sensor configuration.

Sensor	Sensor name	Target Gases
1	TGS 2610 5U	Alcohols/Alkanes
2	TGS 2600	Alcohols/Alkanes
3	TGS 2612 5U	Alkanes
4	TGS 2603	Sulfur/Nitrogen
5	TGS 2602	Nitrogen/Sulfur

Prior to sampling, the NOS.E was turned on and allowed to ambiently sample the environment for 20 min to determine baseline sensor response to the environment. The NOS.E samples were taken prior to dog sampling trials, with constant air intake rate at max capacity (~1.1L/min) as in previous studies [22,24]. Samples were taken in a continuous manner while moving over the grave with the NOS.E air intake system hovering approximately 15 cm above ground, moving from R1 to R6 in numerical direction (1–13; Fig 1). A pre-set path was selected for consistency across sampling days and to ensure all grave regions were covered. Any sensor responses were recorded as time marks and response locations were transcribed on a map of the grave (Fig 1).

The control grave located 5 m away from the experimental grave was also sampled on each trial day to compare NOS.E sensor responses. Environmental responses of the general site area were also recorded to ensure that any VOC presence were not due to the environment.

The NOS.E data recordings were taken continuously by walking over the grave to emulate a search event. When a response from any sensor was observed above baseline, the sampling nozzle was held still until a maximum sensor response remained constant. This event was then recorded as a “hit”. Following the “hit”, the air-intake system was then moved away from the target and the sensors were allowed to return to baseline prior to recommencing sampling across the remaining grave. The NOS.E sampling protocol involved a series of wash steps between each sampling event to eliminate carryover or cross contamination (Table 3). An active VOC sampling time of 200 s per sequence was used and stitched together during data processing to cover the entire grave area.

**Table 3:** NOS.E sampling metrics during VOC sampling.

Chamber Wash I	100s
Vacuum Time I	10s
Baseline Setup	20s
Vacuum Time II	10s
Sample Time	200s
Baseline Recovery	100s
Chamber Wash II	100s

### 2.4 NOS.E data processing

The NOS.E (.txt) files were processed using a peak detection algorithm adapted from Brown *et al*. [22] to accommodate for multiple hits within the same file. This algorithm was intended to automate the calculation of peak heights. All detected peaks were reviewed by the authors to determine validity and inclusion in further analysis. This algorithm was developed using Python 3.7.12, pandas (1.3.5) [35], scipy (1.7.3) [36] and peakutils (1.3.5) [37] with data visualization using matplotlib (3.5.3) [38] and seaborn (0.12.2) [39]. The code, NOS.E data files and output files can be found at github.com/Taphonomic-Analytical-Forensics/nose-dogs.

Briefly, peaks (and hits) were automatically detected using the scipy.find_peaks function with a prominence value set to 0.025. This value was chosen based on a manual assessment of the resulting peak hits to ensure that there were no false negative readings. The normalized peak heights were determined by subtracting the baseline (determined using the peakutils.baseline function with default arguments) from the peak maxima. Peak heights were given as zero for sensors where the difference between the maximum and minimum sensor responses was less than 5% of the minimum response, or where there were no peaks using the peak detection algorithm. Peaks were also aligned across data files to correlate with the hits from the CDDs. To include the environmental data,.csv files were imported into Python and incorporated into the visualization of the NOS.E pre-and post-probing plots. An example of a sampling run showing all five sensor profiles with calculated peak heights can be found in S2 Fig. in the Supplementary Information.

NOS.E sensor responses (Sensors 2–5; Sensor 1 was excluded as there was only one data point) were visually compared to CDD alert type using box-and-whisker plots and violin plots. The combination of low sample sizes and high variables gave this dataset low statistical power and limited rigorous statistical analysis. Spearman’s rank correlation (ρ) was selected to compare NOS.E sensor responses to alert types as an investigatory method. This method was selected as it is better suited for small sample sizes that are not normally distributed, are non-linear, and are monotropic, as observed in this dataset. Missing sensor values were imputed using k-nearest-neighbor (kNN) imputation, where (k = 5). Additionally, to determine whether statistical significance was present under multiple testing events, particularly due to low sample sizes, Benjamini-Hochberg (B-H) false discovery rate (FDR) adjustment was applied to p-values.

### 2.5 GC × GC-TOFMS sample collection

VOC samples for laboratory analysis were collected concurrently with the dog trials and NOS.E sampling days. All samples were taken after probing, with the exception of day 98. Three replicate samples were collected from the headspace of both the test grave and control grave. A large aluminum hood was placed over each grave for 20 min to allow the VOCs to accumulate prior to being collected onto a dual sorbent tube containing Tenax TA and Carbograph 5TD (Markes International Ltd., UK; parts number C2-AAXX-5149). An ACTI-VOC™ low-flow pump (Markes International Ltd., UK) was used to actively sample VOCs for 10 min at a rate of 100 mL/min to collect a total volume of 1 L of headspace. Each tube was then wrapped in aluminum foil and transported in air-tight jars to the laboratory for analysis. Prior to sample analysis, each sorbent tube was injected with an internal standard of 0.2 µL of 10 ppm bromobenzene constituted in methanol (Sigma-Aldrich, Sydney, Australia) using an eVol® XR hand-held automated analytical syringe (SGE Analytical Science, Sydney, Australia).

### 2.6 GC × GC-TOFMS analysis

Each sorbent tube underwent thermal desorption (TD) using a Markes Unity 2 Thermal Desorber (Markes International, Ltd, USA) and a Series 2 ULTRA multi-tube autosampler (Markes International, Ltd, USA). Each tube was desorbed at 300°C for 4 min, before being transferred to a general-purpose cold trap (TenaxTA/Carbograph 1TD) at a flow rate of 25 mL/min to a cold trap held at −15°C. The cold trap desorption was performed at 300°C for 4 min with a flow rate of 25 mL/min.

Sample analysis was performed using a Pegasus 4D GC × GC-TOFMS (LECO, Castle Hill, NSW, Australia) connected to the TD unit using a 1 m uncoated fused-silica transfer line (Markes International Ltd.), heated to 120°C. The TD transfer line was joined to the primary column using an Ultimate Union kit (Agilent Technologies, Mulgrave, VIC, Australia). The primary column was a Rxi®-624Sil MS (30m x 0.250 mm id, 1.40 µm film thickness) and a Stabilwax® (2m x 0.250 mm id, 0.5µm film thickness) (Restek Corporation, Sydney, NSW, Australia) was used as the secondary column. A SilTite^TM^ µ-Union (SGE Analytical Science, Ringwood, VIC, Australia) was used to connect the primary and secondary columns. Helium was utilized as the system carrier gas and maintained at a constant flow rate of 1.0 mL/min. The primary oven was held at 35°C for 5 min before being increased to 240°C at a rate of 5°C/min and then held at 240°C for an additional 5 min, giving a total run time of 51 min. Modulator offset was set at 5°C with the secondary oven temperature offset at 15°C, with a modulation period of 5 s. The mass spectrometer transfer line was kept at 250°C and mass acquisition performed at a range between 29–450amu at 100 spectra/s. Ion source temperature was kept at 200°C and electron ionization energy at −70 eV with a 200 V offset detector voltage.

### 2.7 GC × GC-TOFMS data processing

Data processing was performed using ChromaTOF® software (version 4.51.6.0, LECO, Australia). Baseline tracking was performed with an offset of 80% and automatic baseline smoothing, with a 150 signal-to-noise ratio. A 30 s peak width was used in the first dimension and a 0.15 s peak width was used in the second dimension. The National Institute of Standards and Technology (NIST, 2011) Mass Spectral Library was used with a minimum library match factor of 80% to establish a compound list. To perform compound alignment across samples, the samples were classed into their respective sampling day. All control samples were added to the same class. To be retained, analytes had to be present in 30% of the samples within a class or in two of the samples overall, due to the inherent differences expected between sampling days due to the decomposition process.

Following initial data processing, the compound lists were exported into Microsoft Excel where known contaminants and instrument artefacts (column bleed, solvents, etc.) were removed. Replicate samples were averaged and blank subtraction was done using a 50% threshold whereby the analyte in the experimental samples had to be present in ≥ 50% of the analyte response of the blank [22,31,34]. The remaining compounds were then sorted into their respective compound classes (alcohol, aldehyde, alkane, alkene, aromatic, ester, ether, halogenated, ketone, nitrogen containing, sulfur containing, or other). Data visualization was performed using Python 3.7.6 with seaborn (0.11.1) [39] and matplotlib (3.3.4) [38].

## 3. Results and discussions

### 3.1 Environmental data

The search trials commenced on Day 98 post burial (July 1^st^, 2020), with the average daily temperature during the search days ranging from 9.1–13.7˚C, with a daily average temperature of 13.4 ˚C (Fig 2). This is reflective of an Australian (Sydney) winter, conditions where VOCs are less readily available than during warmer months [29,30]. A large period of heavy rainfall occurred between days 123–125 and between days 135–143 post-burial.

**Fig 2:**
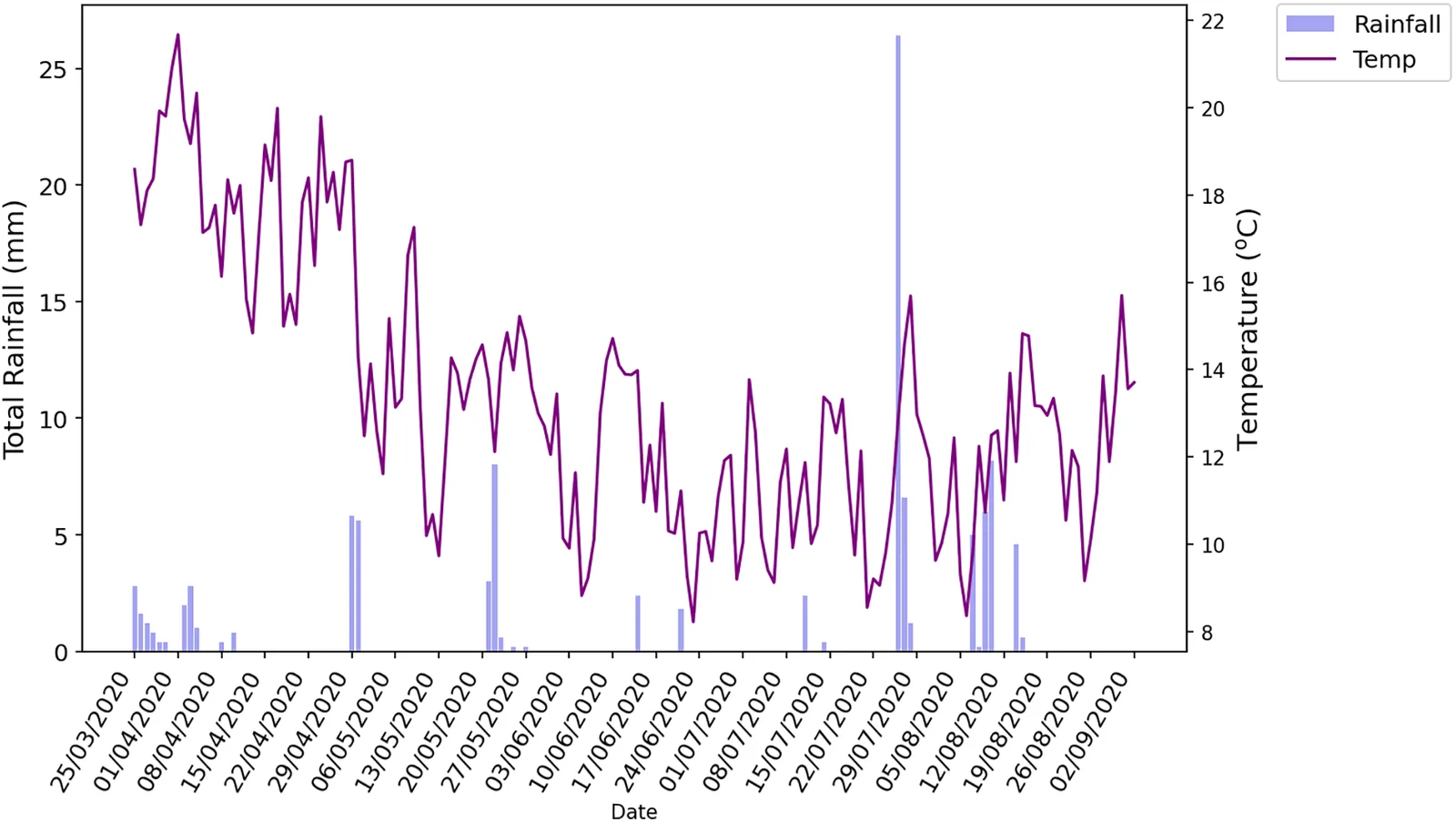
Environmental data including average daily temperature (°C) and total daily rainfall (mm).

### 3.2 NOS.E data

The results from the NOS.E were evaluated for each sampling day (Fig 3) and separated into categories of pre- and post- grave probing. NOS.E responses were recorded on all sampling days. The number and abundance of “hits” (defined as the number of times the NOS.E sensors produced a signal above baseline during the grave sampling) considered as positive responses were higher post-probing. This was expected, as the probing functions to release the VOCs from the burial environment [31], thereby making trapped VOCs available for dispersion and detection. Sensor 2, corresponding to alcohols and alkanes, and Sensor 5, corresponding to nitrogen and sulfur, were the most reactive throughout the trial and were the primary sensors which responded. From previous works, Sensor 5 has higher sensitivity to VOCs, reacting to concentrations 1.5–2 times lower than all other sensors present in the sensor array [24]. Sensor 3 (Alkanes) only reacted on days 126 and 161. Day 161 showed sensor responses from all NOS.E sensors, which were not reported on any other sampling day. On this day during the probing stage, the probe accidentally hit and punctured the buried remains. This resulted in the release of internally trapped VOCs that would not otherwise have been detected. These sensor responses, particularly that of Sensor 1, are therefore not likely to be indicative of VOCs present in a standard search scenario where internal VOCs are contained within the remains and thus is not likely to be a primary sensor type for clandestine grave detection.

**Fig 3:**
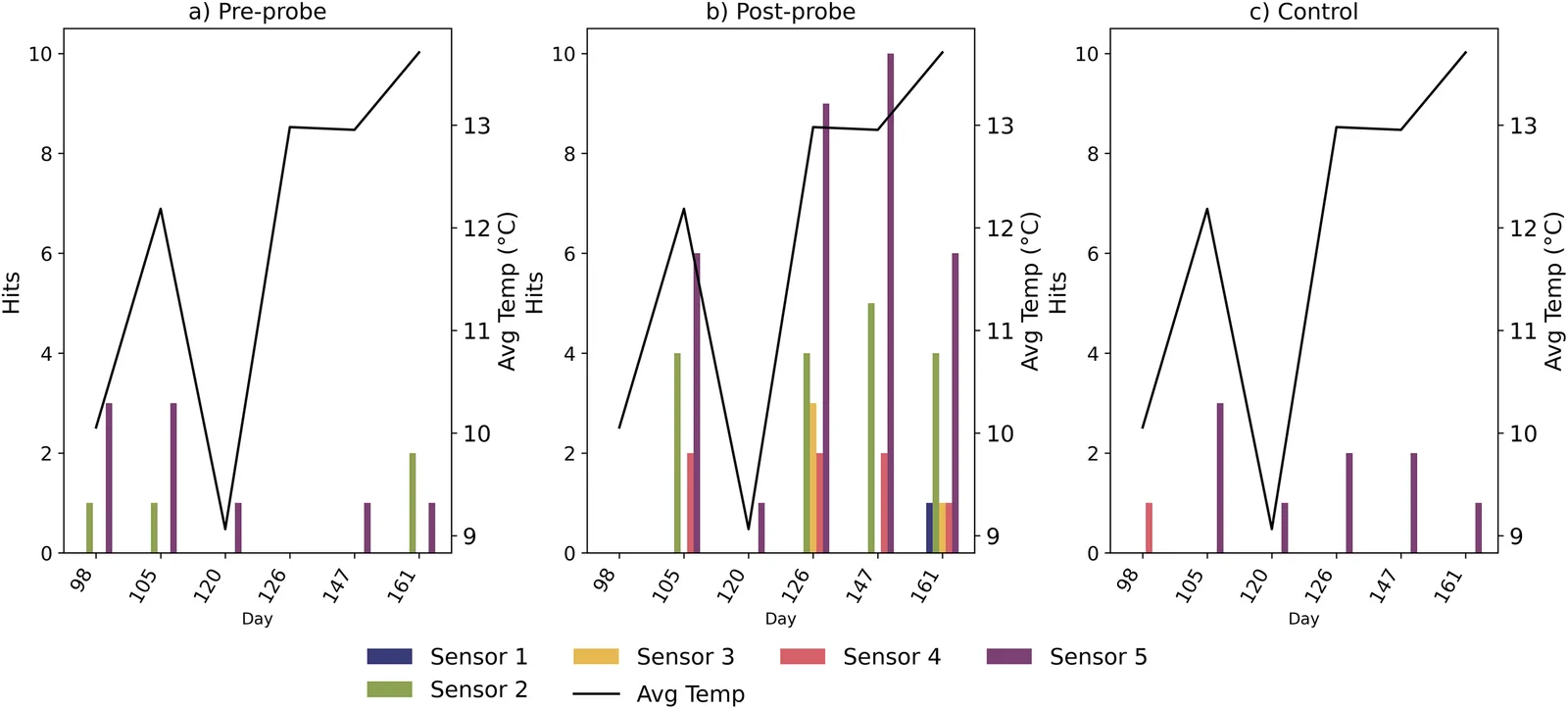
NOS.E sensor responses in relation to temperature changes over sampling days. (a) represents data taken pre-probing of the clandestine grave while (b) represents data taken post-probing and (c) represent readings taken from the control grave. Note: post-probing data was not collected on day 98.

The NOS.E recorded some sensor responses overtop of the control grave. This grave was located upwind and uphill of the buried donor, and 200 meters away from any other cadaver. Due to the location of the control grave, along with NOS.E sampling prior to dog trial runs, it is unlikely that these sensor responses are attributed to decomposition VOC contamination. A majority of responses were recorded from Sensor 5, detecting nitrogen and sulfur-containing compounds. As soil matrices are sinks in both the nitrogen and sulfur cycle, it is likely the sensor responses are attributed to environmental sources of nitrogen and sulfur.

Interestingly, prior to probing, there was no noticeable effect on NOS.E sensor responses from any of the assessed environmental variables (temperature, solar radiation, relative humidity, wind speed, wind direction and rainfall). However, in post-probe scenarios, there was a potential correlation between temperature and increased sensor response for the NOS.E (Fig 3). The limited number of samples prevents statistical analyses of these observations. However, this observation differs to previous work where the NOS.E sensors were additionally influenced by gust speed and wind direction for detecting a human donor placed above ground [22]. It is likely that NOS.E performance differences may be attributed to human remains location (surface vs buried), time of year (summer versus winter) or VOC availability (freely available in air matrixes versus soil interference) which require further testing.

### 3.3 In-field search results

As per the operating protocol of the NSWPF Canine Unit, each of the dogs were directed downwind of the buried donor in order to catch the edges of the scent cone and allowed to search independently for the clandestine grave. To minimize the chance of dogs relying on memory of the grave location rather than using the scent, the dogs were released from different areas of the site each trial day. Each dog alerted their handler by either laying down or barking to confirm their proposed detection of the buried remains. After probing events, all three dogs alerted at holes probed over the upper torso of the remains (Table 4; R1 and R2). Following this, the NOS.E also alerted with the highest sensor readings in this area, likely due to the number and concentration of volatiles emitted from this region due to the presence of multiple decomposing organs [22].

**Table 4:** Cadaver detection dog alert responses and location compared to NOS.E above baseline response and location (reference Fig 1) in-field. N/A indicates that the dog was not a part of the trial for that sampling day.

		Canine trial				NOS.E Trial
Day Postmortem	Probed	Canine	Alert	Alert Classification*	Location of Alert	Location of Alert
**D98**	No	Dog 1	N/A	N/A	N/A	N/A
		Dog 2	Yes	True Positive	R1, partial at R6	R2, R6
		Dog 3	Yes	True Positive	R2, partial at R6	
**D105**	No	Dog 1	Yes	False Positive^†^	R1/R2	–
		Dog 2	N/A	N/A	N/A	
		Dog 3	No	True Negative	–	
	Yes	Dog 1	Yes	True Positive	R1	R2 (multiple hits), R4, R6
		Dog 2	N/A	N/A	N/A	
		Dog 3	Yes	True Positive	R3	
**D120**	No	Dog 1	N/A	N/A	N/A	R3
		Dog 2	No	True Negative	–	
		Dog 3	N/A	N/A	N/A	
	Yes	Dog 1	N/A	N/A	N/A	R3
		Dog 2	Yes	True Positive	R3	
		Dog 3	N/A	N/A	N/A	
**D126**	No	Dog 1	N/A	N/A	N/A	–
		Dog 2	No	True Negative	–	
		Dog 3	No	True Negative	–	
	Yes	Dog 1	N/A	N/A	N/A	R1 (multiple hits), R2, R3, R4
		Dog 2	Yes	True Positive	R1/R2	
		Dog 3	Yes	True Positive	R1/R2	
**D147**	No	Dog 1	N/A	N/A	N/A	R2
		Dog 2	N/A	N/A	N/A	
		Dog 3	No	False Negative	–	
	Yes	Dog 1	N/A	N/A	N/A	R1, R2, R3
		Dog 2	N/A	N/A	N/A	
		Dog 3	Yes	True Positive	R4	
**D161**	No	Dog 1	N/A	N/A	N/A	–
		Dog 2	Yes	False Positive^†^	–	
		Dog 3	N/A	N/A	N/A	
	Yes	Dog 1	N/A	N/A	N/A	R1, R2
		Dog 2	Yes	True Positive	R2	
		Dog 3	N/A	N/A	N/A	

*Relative to NOS.E readings, ^†^Alerted to handler.

As sampling weeks continued, the CDDs continued to alert at the same probed holes in regions R1 and R2 (Fig 1). During the trials where the grave was not re-probed, all three dogs either did not alert (Dog 2 and 3) or provided false positive alerts (Dog 1 and 2). After the graves were re-probed, all dogs were able to locate the grave with positive alerts (Table 4).

The NOS.E was used to assess whether or not compounds were being emitted or if partial profiles were causing false positive alerts. On sampling Day 105, Dog 1 gave a false positive alert prior to probing. The NOS.E did not produce a signal to targeted volatiles in this region prior to probing (Table 4). Dog 3, who was also present during the trial, did not alert prior to probing on Day 105. Dog 3 has significantly more field experience than Dog 1 (Table 1), as Dog 1 only had one year of experience at the time of this study which likely attributed to the false positive alert.

### 3.4 GC×GC-TOFMS results

Compounds identified from the GC × GC-TOFMS analysis were classified into chemical classes based on functional groups and were averaged per sampling day. The identified VOC classes included halogenated compounds, furans, esters, ethers, ketones, aldehydes, alcohols, alkanes, cycloalkanes, alkenes, nitrogen-containing compounds, sulfur-containing compounds, and aromatics (S1 Table). To allow for direct comparison with the NOS.E data, only compound classes that could be detected by the NOS.E sensors were further analyzed in this study.

All of the selected VOC compound classes were detected throughout the entirety of the trial (Fig 4) with an increase in average peak area abundance observed on day 126 and further on day 147. These results were also supported by NOS.E (Fig 3). The increases were likely due to the rehydration of the remains following a period of rainfall which occurred just days before sampling, alongside an increase in the average temperature (Fig 2) which would have promoted decomposition [40,41] resulting in an increase in VOC production.

**Fig 4:**
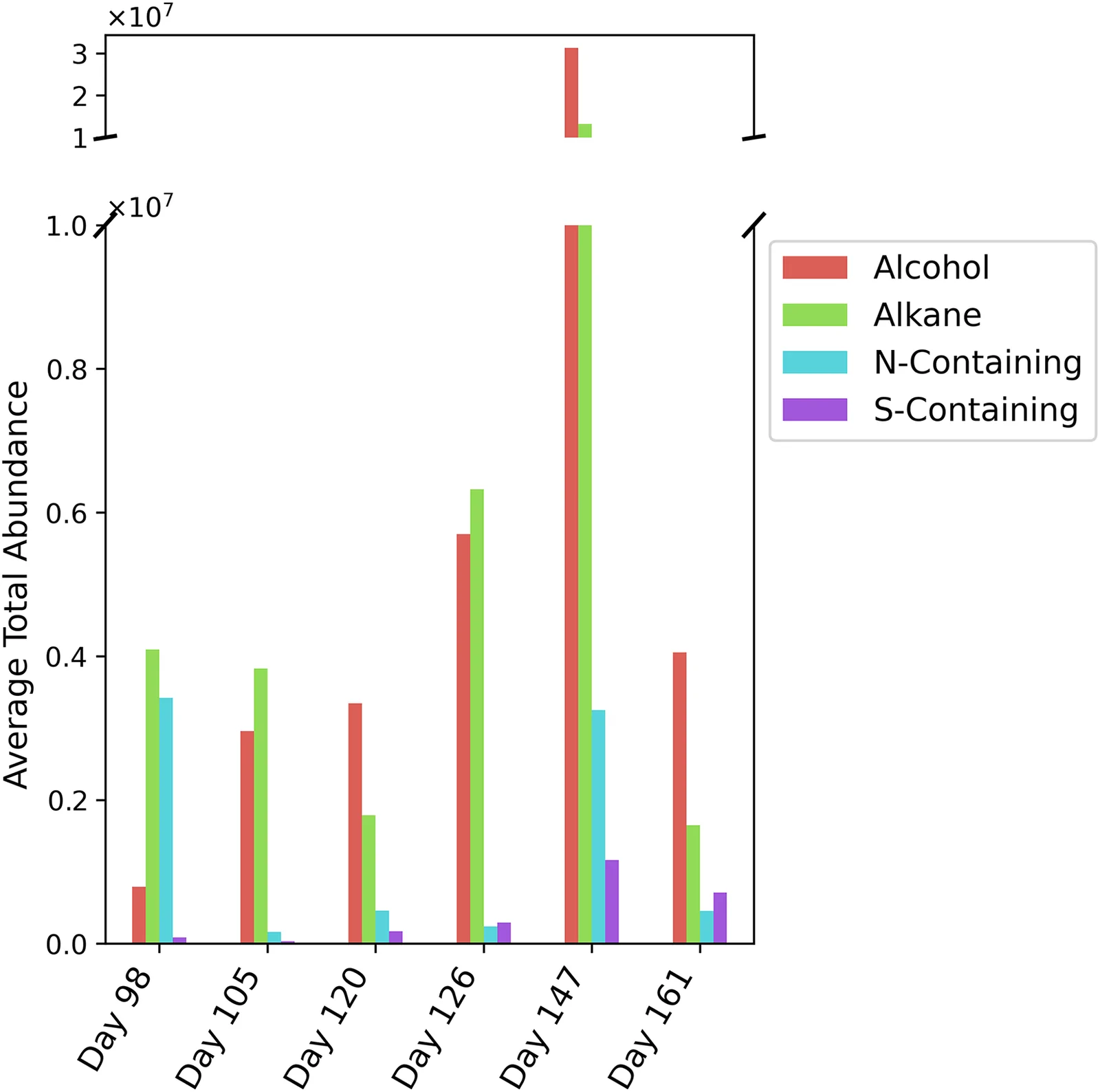
VOC Abundance as peak area (n = 3) summed and averaged from the clandestine burial site over the sampling period. Burial was probed for all days except day 98. Note, only those compound classes detectable by the NOS.E sensors are shown.

Nitrogen containing compounds were detected in higher quantities on days 98 and 147. Sulfur containing compounds also peaked on sampling day 147. However, on the majority of the trial days, alcohol and alkanes were the main contributors to the VOC profiles. Interestingly, Sensor 5 (nitrogen and sulfur containing compounds) had the highest response of all NOS.E sensors across the sampling days, which does not align directly with VOC data. This is likely due to the sensitivity and response profile (*i.e.,* log response curve) of Sensor 5, as reported previously [24]. Sulfur containing compounds are the most widely recognized group detected in decomposition odor [10,17,32,34,42–45]. In this study, dimethyl disulfide (DMDS) was the most consistently observed polysulfide (S1 Table). Several nitrogen containing compounds, commonly found during the early stages of decomposition [6,46], were also detected across sampling days (Fig 4 and S1 Table) but similarly to the sulfur compounds, they appeared in very low abundance during the GC × GC-TOFMS analysis. Alcohols were found to increase during the study and dramatically spiking at Day 147 before rapidly declining on Day 161 (Fig 4). Alkanes followed a similar trend to alcohols, however there was a noticeable decrease in abundance on Day 120.

### 3.5 NOS.E and CDD comparison

NOS.E sensor “hits” were compared to CDD alert responses. There were no false positive alerts from the most experienced dog (Dog 3; Table 4). Additionally, false positive alerts only occurred at times when the grave was not probed (Table 4). Violin plots of all five sensor responses were conducted to compare with alert responses between CDDs that had multiple trials (Fig 5a and 5b). The distribution of true positive hits compared to sensor type response demonstrated that one CDD (Fig 5a) had a wider distribution of true positive alerts in comparison to the CDD that was present at multiple trials (Fig 5b). Additionally, one dog had more resolute alerts when higher concentrations of sulfur or nitrogen containing compounds were present. This may be an artifact of individual differences, breed differences (English Springer Spaniel versus Labrador retriever), or experience (6 years versus 2 years). Both the NOS.E and VOC data demonstrate that the increase in abundance of sulfur and nitrogen containing compounds post-probing may have contributed to Dog 2 correctly producing a true positive alert. The violin plots indicate that the collective response of Sensors 2 (Alcohols/Alkanes), 4 (Sulfur/Nitrogen), and 5 (Nitrogen/Sulfur) are more important for accurate detection than Sensors 1 (Alcohols/Alkanes) and 3 (Alkanes). Observationally, it appears that there is a threshold for discerning human remain VOCs from environmental sulfur, (Sensor 5 (Nitrogen/Sulfur)), as low sensor responses were detected on no alert areas. However, once the sensor response was above a certain peak height, the response aided the dogs in providing true positive alerts. Supporting this, the false positive alert for Dog 2 demonstrated a similar Sensor 5 response to the “No Alert”, but none of the other sensor responses.

**Fig 5:**
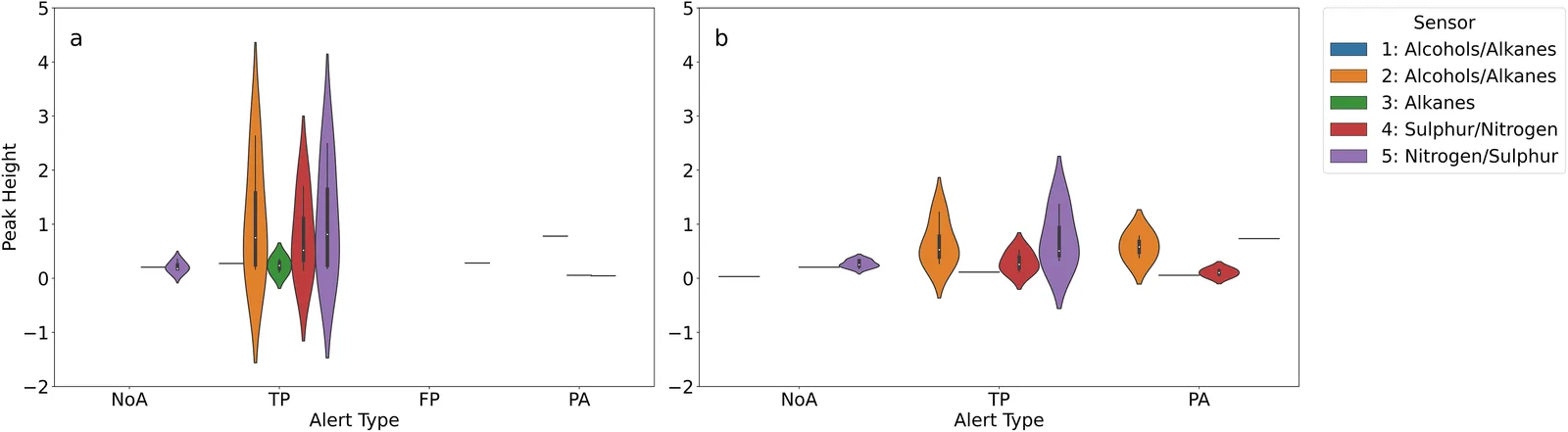
Violin plots of cadaver detection dog (CDD) alerts for CDD 2 (a) and CDD 3 (b). Each sampling point represents a recorded alert. “NoA” represents “no alert”, “TP” represents “True Positive”, “PA” represents “partial alert” and “FP” represents “False Positive.” No “FP” data were recorded by CDD 3.

This assessment is further supported when comparing NOS.E sensor responses to all dog alert types (including Dog 1; Figs 6 and 7). It appears that it is both the presence and threshold of VOC concentration classes that influence true alerts from CDDs to buried human remains.

**Fig 6:**
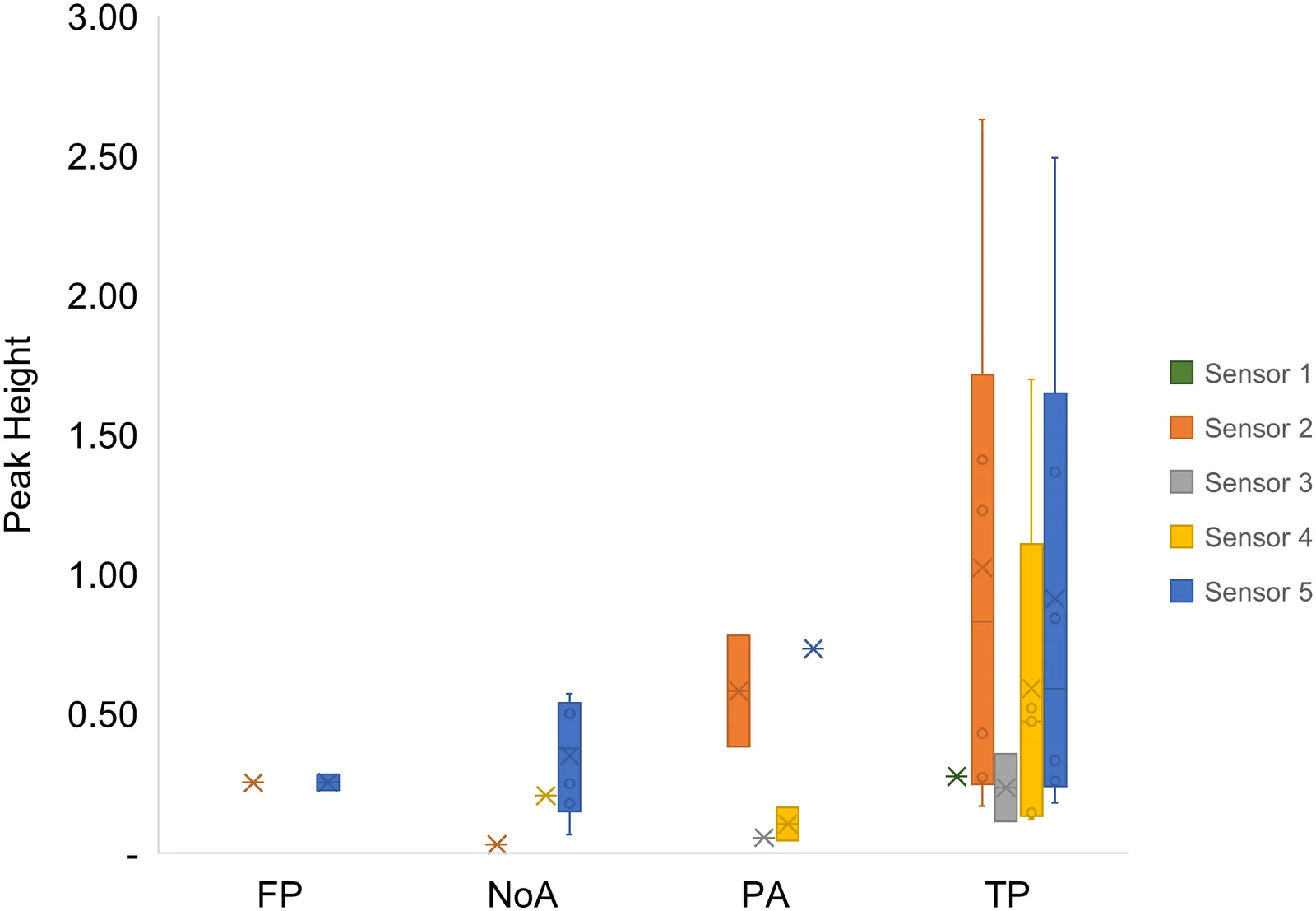
Box and Whisker plots of combined cadaver detection dog (CDD) alerts to the clandestine grave. Each sampling point represents a recorded alert from each location (R1-13) where both the CDD and the NOS.E alerted. “FP” represents “False Positive.”, “NoA” represents “no alert”, “PA” represents “partial alert” and “TP” represents “True Positive”.

**Fig 7:**
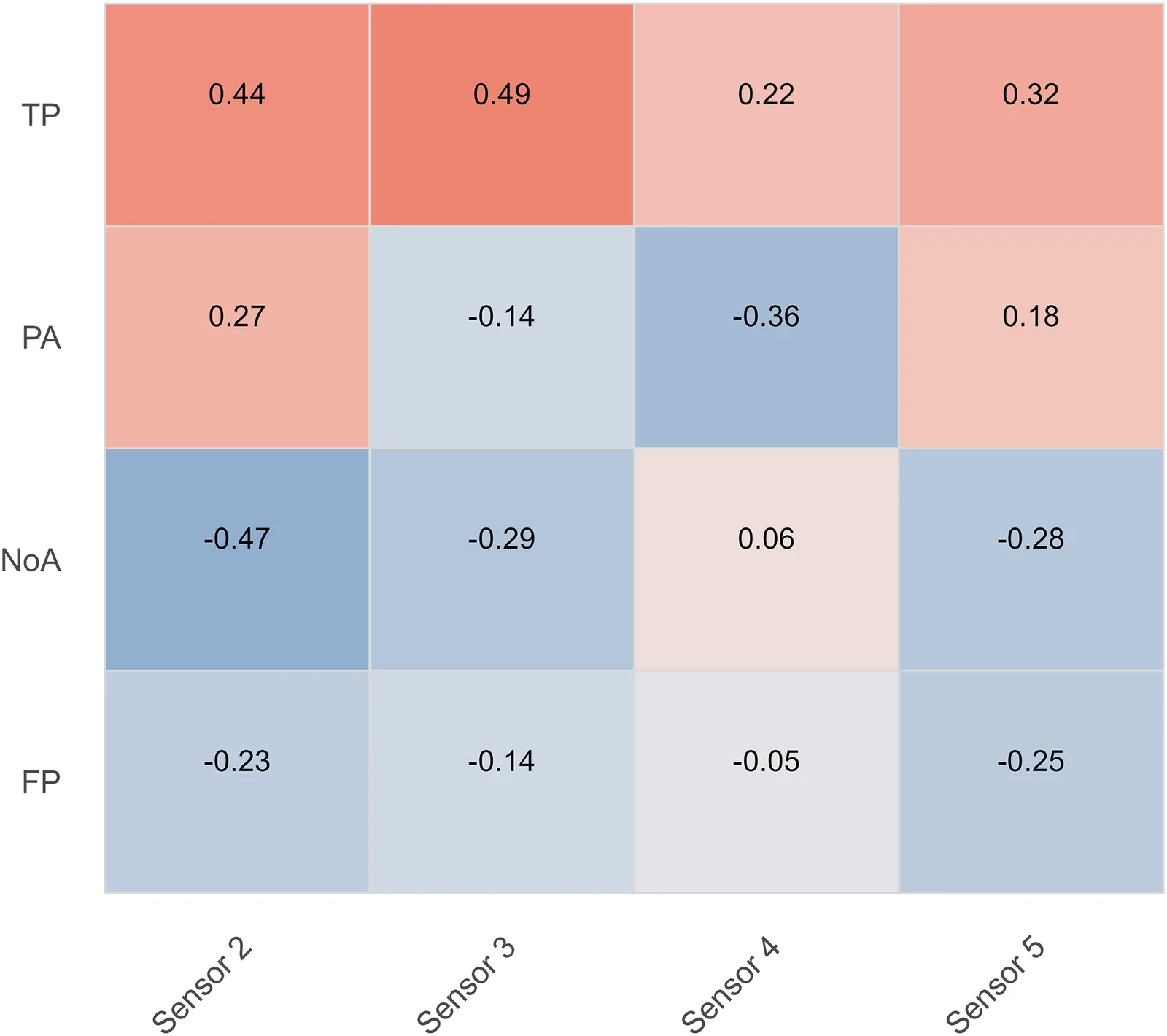
Spearman’s rank (ρ) correlations after kNN imputed data and BH corrections across sensor values and cadaver detection dog (CDD) alert type. “FP” represents “False Positive.”, “NoA” represents “no alert”, “PA” represents “partial alert” and “TP” represents “True Positive”. Tile colors represent the sign and magnitude of ρ using a diverging scale, where red represents a positive association and blue represents a negative association.

Computed Spearman’s rank (ρ) correlations across kNN imputed sensor responses did not show any statistically significant associations between response type and NOS.E sensor response value after controlling for false discovery rate (BH-adjusted p-values). Observationally, ρ values demonstrated positive monotonic associations between true positive alerts and sensor value, while no alerts and false positive demonstrated negative associations. Namely, true positive alerts tended to increase across all higher sensor values, whereas no alerts and false positives decreased as sensor values increased. Partial alerts reflected a mixed pattern to all sensors. However, as these values were not statistically significant, caution should be taken in interpretation.

## 4. Conclusions

This work provided novel insight into how VOC class and concentration impact CDD detection capability in terms of alert responses. Specifically, this work helped discern which VOC compound classes were relevant for true response alerts from CDDs, as well as provided insight into which VOC classes and concentrations were less likely to cause confident responses for CDDs in detecting human remains in a buried environment. Prior to this study, none of the CDDs had exposure to buried human remains. This study was limited, as only one donor could be assessed, and VOC variability associated with age, sex, body mass index, and other environmental factors, such as soil composition, could not be adequately or statistically assessed. However, this pilot work was beneficial in understanding CDD capabilities that have been developed with their current training protocols and forms a foundation for future work incorporating multiple donors and larger-scale studies.

The NOS.E provided similar sensor responses to CDDs, which was later supported by analytical VOC data. As in previous works, the presence of sulfur and nitrogen containing compounds are critical for CDD true positive alerts. This study helped demonstrate that concentration of sulfur and nitrogen containing compounds play a role in true detections. It was determined that probing the grave increased the detection ability of the NOS.E (Fig 5) both in terms of compound class detection and intensity of the hit. Probing the grave also increased the detection accuracy of less experienced CDDs, whereby after probing there were no false positive or false negative alerts (Table 4).

Some variables were unavoidable or uncontrollable in this study, due to the working commitments of the police and the CDDs. For example, there were varying numbers of field tests per dog. Despite alternating schedules, days between trials and alternating the order in which the CDDs searched, there is a potential that the CDDs became familiar with the location of the grave. Therefore, caution should be taken with interpreting lower VOC threshold values for more experienced dogs. However, as there were no false negative alerts after probing, this demonstrates that CDD alerts were more likely due to a change in the scent picture than location memory. To further support this, additional studies with more donor sites concurrently would be beneficial.

Additionally, in order to be field-deployable, additional forensic testing and validation, including sensor detection limits, responses to various soil types and burial depths, and comparisons to mixed odors will need to be conducted. Despite the study limitations, having concurrent analytical data as to what and if the dogs are detecting human decomposition VOCs, is useful in the further training of current and new cadaver detection dogs.

## Supporting information

S1 Fig Flow chart depicting the study objective and methods that were compared. (PDF)

S2 Fig Example sampling run of the grave on day 161 post-probing. The red line is the calculated baseline with the vertical black line the distance from the baseline to the peak maximum.
(PDF)

S1 Table Showing the individually tentatively identified VOCs of the experimental grave per sampling day. Only VOCs that were present in three or more sampling days are presented here.
(PDF)
