# Supplementary material for: A case study comparing the detection ability of cadaver detection dogs and electronic nose technology to a clandestine grave
Source: PLoS One. 2026 Oct 21;21(10):e0360233. doi: 10.1371/journal.pone.0360233 (PMC13686469; doi:10.1371/journal.pone.0360233)
Supplement: Supplementary file 1 [file pone.0360233.s001.pdf]

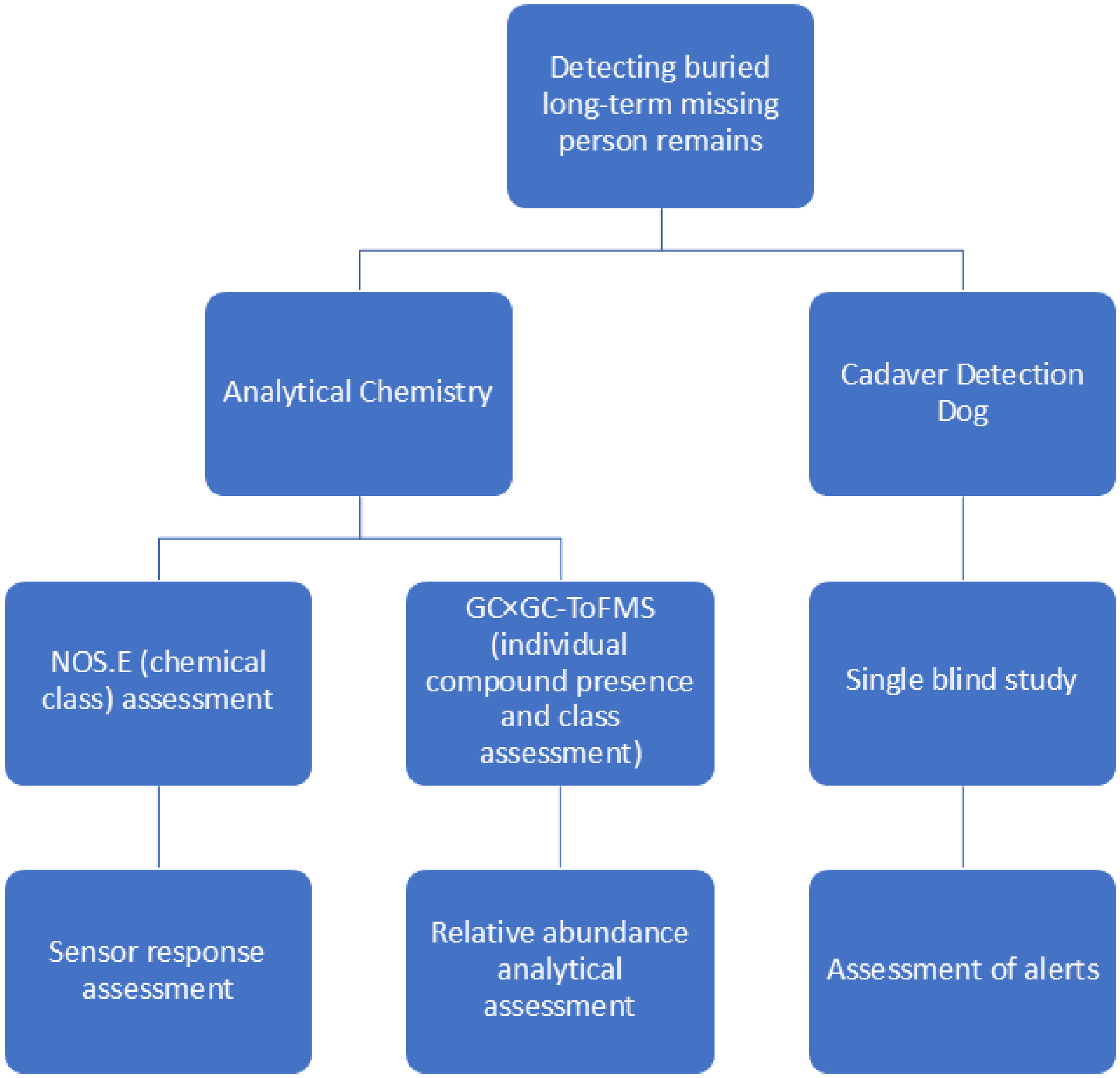

S1 Fig. Flow chart depicting the study objective and methods that were compared.
