# Supplementary material for: A case study comparing the detection ability of cadaver detection dogs and electronic nose technology to a clandestine grave
Source: PLoS One. 2026 Oct 21;21(10):e0360233. doi: 10.1371/journal.pone.0360233 (PMC13686469; doi:10.1371/journal.pone.0360233)
Supplement: Supplementary file 2 [file pone.0360233.s002.pdf]

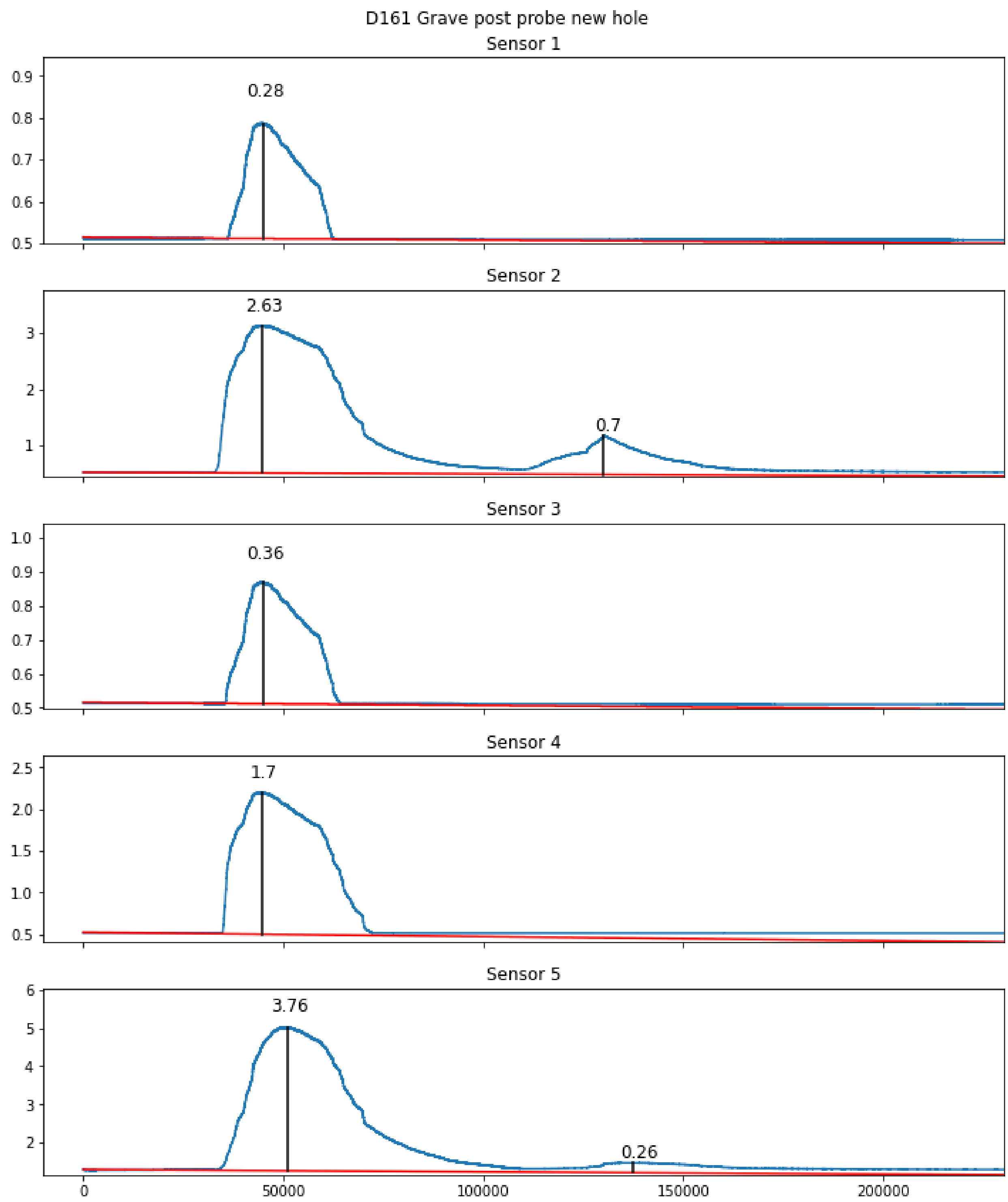

S2 Fig. Example sampling run of the grave on day 161 post-probing. The red line is the calculated baseline with the vertical black line the distance from the baseline to the peak maximum.
