# Supplementary material for: A case study comparing the detection ability of cadaver detection dogs and electronic nose technology to a clandestine grave
Source: PLoS One. 2026 Oct 21;21(10):e0360233. doi: 10.1371/journal.pone.0360233 (PMC13686469; doi:10.1371/journal.pone.0360233)
Supplement: Supplementary file 3 [file pone.0360233.s003.pdf]

S1 Table: Showing the individually tentatively identified VOCs of the experimental grave per sampling day. Only VOCs that were present in three or more sampling days are presented here.

Class	IUPAC Name	Day 98	Day 105	Day 120	Day 126	Day 147	Day 161
**Alcohol**	(2,2,3,3-tetramethylcyclopropyl)methanol	X		X	X		
	3,3,5-trimethylcyclohexanol	X	X	X			
	butan-1-ol			X	X	X	X
	dodecan-1-ol	X	X	X		X	
	ethan-1-ol	X	X	X	X	X	X
	propan-2-ol			X	X	X	X
**Aldehyde**	2-ethylhexanal	X		X		X	
	2-methylprop-2-enal			X	X	X	X
	benzaldehyde	X	X	X		X	X
	butanal			X	X	X	X
	decanal	X		X			X
	dodecanal	X		X		X	X
	ethanal	X	X		X		X
	heptanal	X		X	X	X	X
	hexanal	X		X	X	X	
	nonanal	X		X	X	X	X
	octanal	X		X	X	X	
	pentanal	X	X	X	X	X	X
	prop-2-enal	X		X	X		X
	propanal	X	X	X			X
**Alkane**	2,2,3,3-tetramethylbutane	X	X	X	X		
	2,2,4,6,6-pentamethylheptane		X			X	
	2,2-dimethylbutane		X		X		X
	2,3,4-trimethylpentane	X	X		X	X	X
	2,3-dimethylpentane	X	X		X		
	2,4-dimethylhexane	X	X		X		

	2,5-dimethyldecane	X		X	X		
	2,6-dimethyloctane	X	X	X	X		
	2-methylbutane	X			X	X	X
	2-methyldecane	X	X	X	X		
	2-methylpentane	X	X		X	X	X
	3-ethyl-2-methylpentane	X	X		X		
	3-methyldecane	X	X		X		
	3-methylheptadecane	X	X	X			
	3-methylhexane	X	X		X	X	X
	3-methyloctane	X	X		X		
	5-methyldecane	X	X			X	X
	6,9-dimethyltetradecane	X	X	X			
	6-methyloctadecane	X	X	X			
	butane	X	X		X		
	decane		X			X	X
	dodecane		X	X		X	
	eicosane	X	X	X	X		
	heptane	X	X		X	X	
	hexadecane	X	X	X		X	
	hexane	X	X		X	X	X
	nonadecane	X	X	X	X		X
	nonane	X	X			X	X
	octane	X	X		X	X	X
	pentane	X	X	X	X	X	X
	tetradecane	X	X	X			
	tridecane		X	X		X	
	undecane		X	X	X	X	X
Alkene	1-isopropyl-4-methylenebicyclo[3.1.0]hex-2-ene	X		X	X	X	X
	1-methylcycloprop-1-ene	X	X		X		
	2-methylprop-1-ene	X	X		X	X	X

	3,3,5-trimethylcyclohex-1-ene	X	X	X		X	X
	3,4-dimethylpent-2-ene	X	X	X	X		X
	3,5,5-trimethylcyclohex-1-ene	X	X	X		X	
	5,5-dimethylhexa-1,3-diene	X		X	X		
	but-2-ene	X			X	X	X
	cyclohex-1-ene	X	X	X			X
	hept-1-ene	X	X		X		
	hept-2-ene		X	X	X		
	hept-3-ene	X	X	X	X		
	oct-1-ene	X		X	X	X	X
	octa-1,3-diene	X	X		X		
	pent-2-ene		X	X	X	X	
Aromatic Hydrocarbon	(1-(1-methylpropyl)benzene)	X	X		X		
	1-(1-methylundecyl)benzene	X	X	X	X		
	1-(1-pentylheptyl)benzene	X		X		X	X
	1-(1-propyloctyl)benzene	X	X	X		X	X
	1,3-dichlorobenzene	X	X	X			
	1,6-dimethyl-4-(propan-2-yl)naphthalene	X		X	X		
	1-ethyl-3-methylbenzene	X	X			X	X
	1-methyl-3-(propan-2-yl)benzene	X	X	X	X		
	1-methyl-4-(1-methylethenyl)benzene (p-cymene)	X		X		X	X
	2-ethenylnaphthalene	X	X	X			
	4-ethyl-1,2-dimethylbenzene		X			X	X
	benzene	X	X		X	X	X
	benzothiazole		X	X			X
	cyclohexylbenzene	X		X			X
	methylbenzene (toluene)	X	X			X	X
	Naphthalene	X				X	X
	phenylacetylene				X	X	X
	propylbenzene	X	X			X	

Carboxylic acid	(4-hydroxyphenyl)phosphonic acid	X	X	X	X		
Cyclic Alkane	1,4-dimethylcyclohexane	X	X		X		
	1-ethyl-2-methylcyclopropane	X	X		X	X	X
	cyclododecane	X	X	X			X
	cyclohexane	X			X	X	X
	ethylcyclohexane	X	X		X		
	ethylcyclopropane	X	X	X	X	X	X
	hexylcyclohexane	X	X	X			
	methylcyclohexane	X	X		X	X	X
	methylcyclopentane	X	X		X	X	
Ester	1-methoxypropan-2-yl acetate	X	X			X	X
	2,2,4-trimethyl-1,3-pentanediol diisobutyrate	X		X		X	X
	2-ethylhexyl 2-hydroxybenzoate		X			X	X
	2-ethylhexyl benzoate	X	X	X			X
	3-hydroxy-2,2,4-trimethylpentyl 2-methylpropanoate	X	X	X		X	X
	butyl ethanoate	X	X	X	X	X	X
	dimethyl carbonate		X	X	X	X	X
	dodecyl acrylate	X	X	X	X		X
	methyl benzoate		X	X	X	X	X
	methyl butanoate	X	X	X	X	X	X
	methyl hexadecanoate	X	X		X		
Ether	1-methoxypropan-2-ol	X				X	X
	2-(butoxy)ethan-1-ol	X		X		X	X
	2-ethylhexan-1-ol	X		X		X	X
	6-phenyl-4-(1-phenylethoxy)hex-1-ene	X	X	X			
	di-n-octyl ether	X		X	X		
Furan	2-(butyl)furan	X	X	X			
	2-methylfuran	X			X	X	X

	furan	X			X		X
Halogenated	1,1,1-trifluoropropan-2-one	X		X	X		X
	1,1,2-trichloro-1,2,2-trifluoroethane	X		X	X		X
	1,1-dichloro-1-fluoroethane	X			X	X	X
	1,2,3-trifluoro-5-(trifluoromethyl)benzene	X		X	X		
	1,3,5-trifluorobenzene	X		X	X	X	X
	2-bromotetradecane	X	X	X			
	carbon tetrachloride		X	X	X		
	chlorobenzene	X	X			X	
	trichloromethane	X			X	X	X
	Trichloromonofluoromethane	X		X	X	X	X
	1-chloro-2-methylbutane	X	X		X		
Ketone	1-phenylethan-1-one	X		X		X	X
	2,4-dimethylpent-1-en-3-one	X	X	X	X	X	X
	2,4-dimethylpentan-3-one	X	X	X		X	X
	3-methylbutan-2-one	X			X	X	X
	3-penten-2-one	X	X				X
	4-hydroxy-3-propylhexan-2-one	X		X	X		
	4-methylpentan-2-one	X	X	X	X	X	X
	6-methylhept-5-en-2-one	X	X	X	X	X	X
	but-3-en-2-one	X			X	X	
	butan-2-one	X		X	X	X	
	butane-2,3-dione			X		X	X
	decan-2-one	X		X	X	X	X
	diphenylmethanone	X		X	X	X	X
	heptan-2-one	X		X		X	X
	heptan-3-one	X		X	X	X	X
	nonan-2-one	X	X	X	X	X	X
	oct-1-en-3-one	X	X	X	X	X	X
	octan-2-one	X		X	X	X	X

	octan-3-one	X	X	X	X	X	X
	pent-1-en-3-one	X			X	X	X
	pentan-2-one	X		X	X	X	X
	undecan-2-one	X		X		X	X
N-Containing	2,5-dimethyl-4-nitrohexan-3-one	X	X	X		X	X
	benzonitrile	X		X		X	X
	ethanenitrile	X		X		X	X
	methyl isocyanide	X	X		X		
S-Containing	aminomethanesulfonic acid	X	X				X
	carbon disulfide			X	X	X	X
	dimethyl disulfide		X	X	X	X	X
	dodecyl pentyl sulfite	X	X	X	X		
	sulfur dioxide				X	X	X
Terpene	6,9-guaiadene		X	X	X		
	aristolene	X	X	X	X		
	aromandendrene		X	X	X	X	
	calamenene		X	X	X	X	X
	camphene	X	X	X	X		
	copaene		X	X	X	X	X
	eucalyptol	X	X	X	X	X	X
	isoledene	X	X	X	X		
	ledene		X	X	X		
	limonene	X		X	X	X	X
	α-gurjunene	X	X		X		
	α-phellandrene	X			X		X
	α-pinene	X	X	X	X	X	X
	α-terpineol		X	X			X
	α-terpinolene	X		X	X		
	β-pinene	X		X	X		
